# Discrete Correlation Summation Clustering Reveals Differential Regulation of Liver Metabolism by Thrombospondin-1 in Low-Fat and High-Fat Diet-Fed Mice

**DOI:** 10.3390/metabo12111036

**Published:** 2022-10-28

**Authors:** Steven M. Bronson, Brian Westwood, Katherine L. Cook, Nancy J. Emenaker, Mark C. Chappell, David D. Roberts, David R. Soto-Pantoja

**Affiliations:** 1 Section of Molecular Medicine, Department of Internal Medicine, Wake Forest School of Medicine, Winston-Salem, NC 27101, USA; 2 Section of Comparative Medicine, Department of Pathology, Wake Forest School of Medicine, Winston-Salem, NC 27157, USA; 3 Department of Surgery, Hypertension & Vascular Research Center, Wake Forest School of Medicine, Winston-Salem, NC 27101, USA; 4 Department of Cancer Biology, Wake Forest School of Medicine, Winston-Salem, NC 27101, USA; 5 Atrium Health Wake Forest Baptist Comprehensive Cancer Center, Winston-Salem, NC 27101, USA; 6 Nutritional Science Research Group, Division of Cancer Prevention, National Cancer Institute, National Institutes of Health, Bethesda, MD 20892, USA; 7 Laboratory of Pathology, Center for Cancer Research, National Cancer Institute, National Institutes of Health, Bethesda, MD 20892, USA

**Keywords:** discrete correlation summation, thrombospondin, diet, non-alcoholic fatty liver disease, liver, oxidative stress

## Abstract

Thrombospondin-1 (TSP1) is a matricellular protein with many important roles in mediating carcinogenesis, fibrosis, leukocyte recruitment, and metabolism. We have previously shown a role of diet in the absence of TSP1 in liver metabolism in the context of a colorectal cancer model. However, the metabolic implications of TSP1 regulation by diet in the liver metabolism are currently understudied. Therefore Discrete correlation summation (DCS) was used to re-interrogate data and determine the metabolic alterations of TSP1 deficiency in the liver, providing new insights into the role of TSP1 in liver injury and the progression of liver pathologies such as nonalcoholic fatty liver disease (NAFLD). DCS analysis provides a straightforward approach to rank covariance and data clustering when analyzing complex data sets. Using this approach, our previous liver metabolite data was re-analyzed by comparing wild-type (WT) and Thrombospondin-1 null (*Thbs1^−/−^*) mice, identifying changes driven by genotype and diet. Principal component analysis showed clustering of animals by genotype regardless of diet, indicating that TSP1 deficiency alters metabolite handling in the liver. High-fat diet consumption significantly altered over 150 metabolites in the *Thbs1^−/−^* livers versus approximately 90 in the wild-type livers, most involved in amino acid metabolism. The absence of *Thbs1* differentially regulated tryptophan and tricarboxylic acid cycle metabolites implicated in the progression of NAFLD. Overall, the lack of *Thbs1* caused a significant shift in liver metabolism with potential implications for liver injury and the progression of NAFLD.

## 1. Summary

Thrombospondin-1 (TSP1) is a matricellular protein that regulates cell-to-cell and cell-to-matrix interactions by modulating signaling in response to extracellular factors and through direct interaction with high-affinity cell surface receptors [1]. TSP1 functions as an immunomodulatory molecule that is upregulated in inflammatory conditions. It activates latent transforming growth factor beta-1 (TGF-β) and can lead to the recruitment of leukocytes to inflammatory sites [2,3,4,5,6]. TSP1 is secreted from endothelial cells, monocytes, platelets, and adipocytes. TSP1 secreted from adipocytes functions as an adipokine, and elevated levels of TSP1 are associated with obesity and insulin resistance [7,8]. Therefore, it is not surprising that increased TSP1 levels are implicated in inflammation and metabolic syndrome. Still, the role of TSP1 in the regulation of cellular energetics is not entirely understood.

TSP1 expression also limits carcinogenesis by inhibiting angiogenesis, preventing tumor neovascularization, and affecting tumor cell adhesion, invasion, and proliferation [9]. Loss of TSP1 contributes to increased tumor multiplicity and decreased survival time in a murine model of colon carcinogenesis [10]. We have previously demonstrated that the absence of TSP1 modulates liver metabolism in a C57BL/6J-ApcMin/j (*Apc^Min^*) model of colorectal cancer [10]. To determine the effects of TSP1 in this model, *Apc^Min/+^* mice were crossed with *Thbs1^−/−^* mice to produce the *Thbs1^+/−^*:*Apc^Min/+^* strain. These mice were then crossed with *Thbs1^−/−^* mice to produce *Thbs1^−/−^ Apc^Min/+^* mice. Mice were pair-fed a 5% low fat or 21% high-fat diet starting at weaning [10]. *Thbs1^−/−^:Apc^Min/+^* mice had reduced survival times compared to *Apc^Min/+^* mice when fed a low-fat diet, whereas animals fed a high-fat diet had similar survival times regardless of TSP1 expression. *Thbs1^−/−^* animals had an overall increase in tumor multiplicity, with the animals fed a high-fat diet having significantly more tumors than those fed a low-fat diet. The absence of TSP1 in the context of *Apc^Min/+^* also regulated amino acid and lipid metabolism and eicosanoids and ketone body formation in the liver [10].

The liver plays a significant role in regulating carbohydrate, lipid, and protein metabolism, with diet playing an essential role in the initiation and progression of nonalcoholic fatty liver disease (NAFLD) [11]. NAFLD can progress to nonalcoholic steatohepatitis (NASH) when fat accumulation in the liver causes damage and the onset of inflammation [12]. Lipid metabolism involves several pathways for the breakdown and generation of fatty acids. Complex lipids from the diet are broken down into non-esterified fatty acids, enter the cell by fatty acid transport proteins or diffusion across membranes, and are oxidized in the mitochondria or peroxisomes [13]. In addition, de novo lipogenesis occurs in hepatocytes by converting carbohydrates into fatty acids, exported through lipoprotein production [13]. Fatty acids and derivatives act as ligands and nuclear factors regulating gene transcription and driving liver metabolism by inducing changes in the activity of transcription factors in the peroxisome proliferator (PPAR) [14,15], liver x receptor (LXR) [16,17], hepatic nuclear factor 4 (HNF-4) [18], and sterol regulatory element-binding protein (SREBP) families [19,20,21].

The mechanism of fatty acid regulation in the liver is relatively well understood, but no pharmaceuticals are currently approved to prevent or treat NAFLD/NASH [22]. Preclinical NASH models implicate TSP1 in the modulation of NAFLD/NASH with evidence that *Thbs1^−/−^* mice fed a choline-deficient L-amino acid-defined high-fat diet were protected from the liver damage associated with NASH [12]. Furthermore, plasma TSP1 expression is increased in NAFLD human patients and reduced after fat-lowering lifestyle intervention, which is consistent with the ability of TSP1 to inhibit the uptake of free fatty acids via CD36 in endothelial cells [23,24]. In the same study, treatment with recombinant TSP1 or using a peptide mimetic resulted in the reduced accumulation of fat in hepatocytes mediated through CD36 [23].

Our previous studies examining liver metabolism in a *Thbs1^−/−^*:*Apc^Min/+^* colorectal mouse model focused on the effects of diet and TSP1 in carcinogenesis [10]. Given the paradoxical impact of TSP1 in liver fat accumulation, we reexamined our global metabolomics study focusing on WT and *Thbs1^−/−^* mice fed a low-fat and a high-fat diet to further understand the role of this matricellular protein in liver metabolism and its potential implications in liver injury and diseases such as NAFLD/NASH.

## 2. Data Description

**Figure 1:**
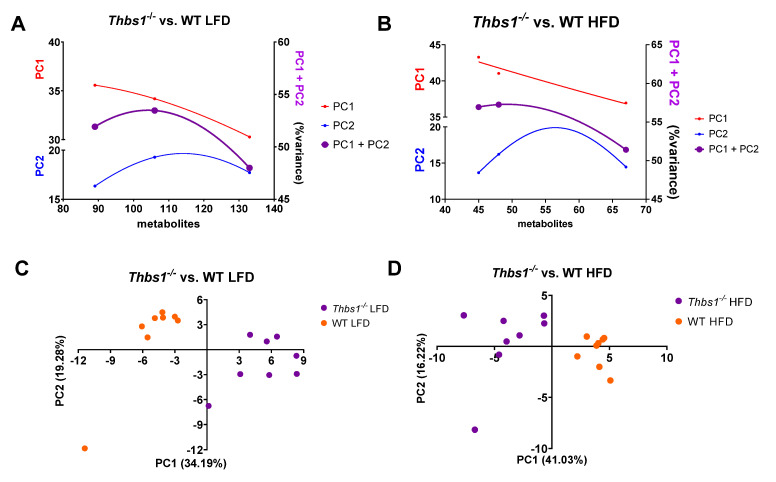
Deficiency of TSP1 is associated with distinct liver metabolic signatures in low-fat and high-fat-diet-fed mice. Discrete correlate summation analysis was calculated to enrich metabolite sets together with those exhibiting a fold change *p*-value of < 0.05 from *Thbs1^−/−^* vs. WT mice fed a low-fat (**A**) or a high-fat diet (**B**). (**C**) Principal component analysis of liver metabolites from *Thbs1^−/−^* vs. WT animals fed a low-fat and (**D**) high-fat diet. N = 8/group.

**Figure A1:**
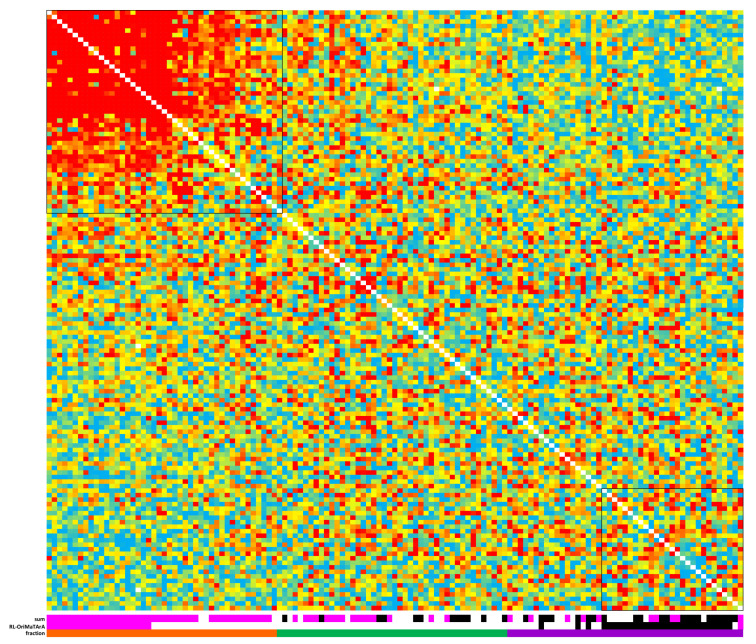
Clustering of the logcr matrix of metabolites from WT and *Thbs1^−/−^* mice fed a low-fat diet with arrays indicating column sums, RL-OriMaTArA, and fractional thirds. Matrix coloring [red > 90th percentile, yellow = 50th percentile, blue < 10th percentile]; sum and RL-OriMaTArA arrays coloring [magenta > Q2, Q2 > white > Q1, black < Q1]; fraction coloring [orange < 0.333, 0.333 < green < 0.667, purple > 0.667].

**Figure A2:**
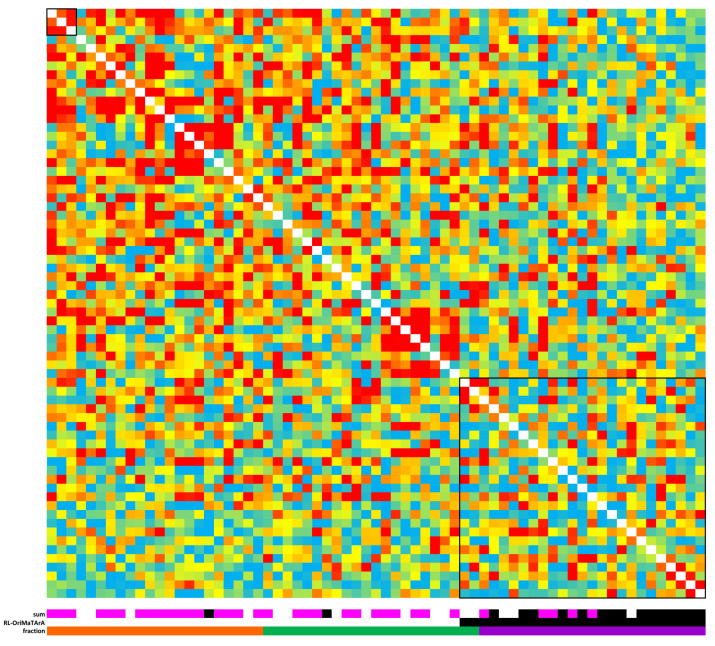
Clustering of the logcr matrix of metabolites from WT and *Thbs1^−/−^* mice fed a high-fat diet with arrays indicating column sums, RL-OriMaTArA and fractional thirds. Matrix coloring [red > 90th percentile, yellow = 50th percentile, blue < 10th percentile]; sum and RL-OriMaTArA arrays coloring [magenta > Q2, Q2 > white > Q1, black < Q1]; fraction coloring [orange < 0.333, 0.333 < green < 0.667, purple > 0.667].

*Thbs1^−/−^* **mice liver metabolites cluster differently than WT liver metabolites.** To determine whether the lack of TSP1 gene expression results in metabolite variance, we reanalyzed metabolomics data from our previous study and compared WT and the *Thbs1^−/−^* mice [10]. Mice were pair-fed a 5% low-fat diet or a 21% high-fat diet at weaning and maintained until 12 weeks of age [10]. At the end of the study, mice were sacrificed, and liver tissue was subjected to metabolomics analysis [10]. Following log transformation and imputation of missing values, ANOVA contrasts were used to identify biochemicals that differed significantly between experimental groups. Metabolite variance was examined using PCA plots (Figure 1A,B); the leftmost points represent PCA variances for all metabolites with a *p*-value less than 0.05, and the rightmost points represent all metabolites with a *p*-value less than 0.1. Discrete correlate summation analysis (DCS) was applied to all metabolites using unadjusted *p*-values. The unadjusted (*p*-values) were used due to the relative number of metabolites that were found to be significantly regulated in our data set. Still when considering standards for reporting metabolomics data [25], we calculated adjusted (q values) which are found in the original dataset [10] and in Supplementary Tables S1–S4. The midpoints represent all metabolites with a *p*-value less than 0.05 plus metabolites clustered by DCS. PCA (Figure 1C,D) was used on the midpoint metabolite sets with an overall (PC1 + PC2) percent variance increase, combined with an enrichment of key metabolites, which would not have been considered with the simple 0.05 *p*-value cut-off. Clustering of the matrices of metabolites from WT and *Thbs1^−/−^* mice fed a low-fat diet (Figure 1A and Appendix A Figure A1) and mice fed a high-fat diet (Figure 1B and Appendix A Figure A2) illustrates the aggregation of DCS metabolites. Principal component analysis indicated that liver metabolites of *Thbs1^−/−^* mice fed a low-fat diet cluster differently than WT mice on the same diet (Figure 1C). This indicates that some metabolic differences segregate mice by genotype. Similarly, analysis of liver metabolites from mice fed a high-fat diet revealed a clear clustering of metabolites between WT and *Thbs1^−/−^* mice (Figure 1D). This suggests that the absence of *Thbs1* gene expression impacts metabolism during basal conditions and after feeding mice a high-fat diet.

**Figure 2:**
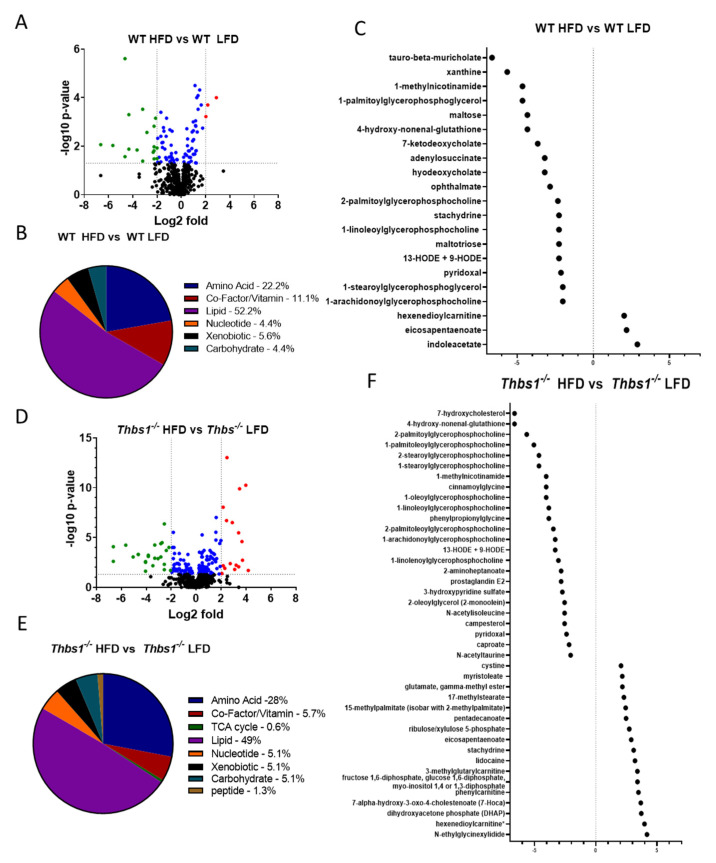
Liver metabolism alterations by diet. (**A**) Volcano plot showing the number of metabolites regulated by diet in wild-type (WT) mice. Downregulated < −2-fold in green; upregulated > 2-fold in red; significantly altered between −2 and 2 in blue. (**B**) Pie chart depicting metabolites that exhibited significant diet-dependent changes in levels in WT mice. (**C**) Dot plot showing significantly regulated metabolites < −2- and > 2-fold in WT mice. (**D**) The volcano plot shows the number of metabolites regulated by diet in *Thbs1^−/−^* mice. Downregulated < −2-fold in green; upregulated > 2-fold in red; significantly altered between −2 and 2 in blue. (**E**) Pie chart depicting metabolites that exhibited significant diet-dependent changes in levels in *Thbs1^−/−^* mice. (**F**) Dot plot showing significantly regulated metabolites < −2- and > 2-fold in *Thbs1^−/−^* mice. N = 8 in each group.

**Figure 3:**
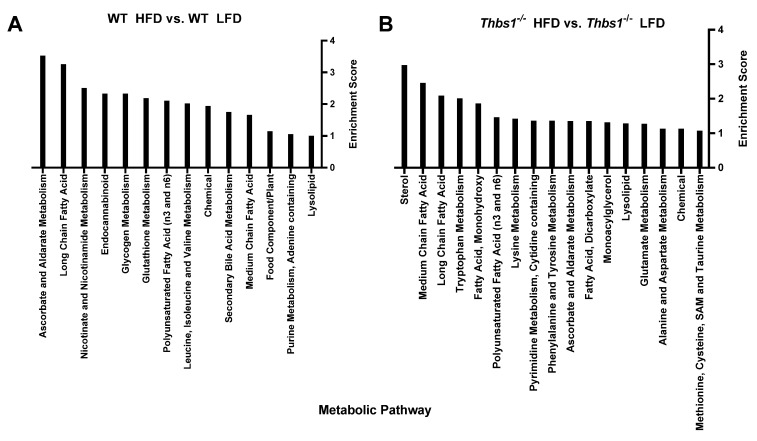
Metabolic pathway enrichment analysis. (**A**) Comparing metabolic pathways regulated by diet in wild-type (WT) mice. (**B**) Comparing metabolic pathways regulated by diet in thrombospondin 1-deficient (*Thbs1^−/−^*) mice.

**Liver metabolite alterations by consumption of a high-fat diet.** Consumption of a high-fat diet in WT mice led to significant (*p* < 0.05) differential expression of 90 metabolites (Figure 2A,B, Supplemental Table S1). Forty-one metabolites were upregulated, and forty-nine were downregulated after feeding a high-fat diet to WT mice. The majority of regulated metabolites (52.2%) were lipid metabolites (Figure 2B). In WT mice, a high-fat diet was associated with hepatic accumulation of various long-chain, saturated, monounsaturated, and polyunsaturated free fatty acids (Figure 2C). Consistently, pathway enrichment analysis of WT HFD vs. WT LFD showed high enrichment of long-chain fatty acids; however, the highest enrichment was observed in ascorbate and aldarate metabolism, suggesting changes in the processing of vitamins and cofactors (Figure 3A). Overall, these data suggest expected metabolic handling when feeding a high-fat diet. On the other hand, providing a high-fat diet to *Thbs1^−/−^* mice resulted in broader alterations in 156 significantly regulated metabolites (Figure 2D), suggesting altered handling of a high-fat diet between WT and *Thbs1^−/−^* animals. As seen with WT, most of those altered (49%) are lipid metabolites (Figure 2E). Pathway enrichment also suggested that the most-regulated pathways include sterol, medium-, and long-chain amino acids (Figure 3B). Although *Thbs1^−/−^* animals had more altered metabolites, the overall percentage of regulated metabolites is similar between groups. In addition to homeostasis and lipid metabolism, converting cholesterol to bile acids is a critical liver function, and NAFLD has been shown to alter bile acid production [26]. WT mice fed a high-fat diet showed a significant reduction of the bile acid tauro-beta-muricholate relative to WT mice fed a low-fat diet, while *Thbs1^−/−^* mice fed a high-fat diet had increased tauro-beta muricholate. This metabolite is known to regulate the Farnesoid X receptor (FXR) superfamily and plays a vital role in the action of bile acids on liver homeostasis [27,28]. *Thbs1^−/−^* mice fed a high-fat diet showed increased beta-muricholate, implicated in liver steatoses [29]. *Thbs1^−/−^* mice fed a high-fat diet had increased levels of cholesterol, as well as the cholesterol precursor squalene and the primary bile acid precursor 7α-hydroxy-3-oxo-4-cholestenoate (7-Hoca) when compared to *Thbs1^−/−^* mice fed a low-fat diet, suggesting increased bile acid synthesis in these animals (Figure 2F and Supplementary Table S2). The next most-regulated metabolic pathway was amino acid metabolism, with about 22.2% of metabolites regulated in WT livers belonging to the amino acid category vs. 28% in *Thbs1^−/−^* livers (Figure 2B,E). According to our analysis, tryptophan metabolism also played a crucial role in developing NAFLD and was one of the top enriched pathways (Figure 3A,B). Serotonin is synthesized from tryptophan; inhibiting its synthesis protects mice from NAFLD by inhibiting obesity [30]. One key difference is that indole acetate was the most elevated metabolite in the WT group (Figure 2C) but was found at lower levels in *Thbs1^−/−^* mice (2.89 log2-fold in WT vs. 0.61 log2-fold in *Thbs1^−/−^*). Indole acetate derived from dietary tryptophan is protective in NAFLD disease [31], suggesting that altered tryptophan metabolism plays a role in disease pathogenesis.

A high-fat diet is associated with oxidative stress [32], another component of the pathogenesis of NAFLD [32,33]. Glutathione is an important antioxidant highly concentrated in the liver [34]. Cysteine-glutathione disulfide, a rate-limiting precursor of glutathione synthesis, was significantly elevated in WT mice fed a high-fat diet compared to *Thbs1^−/−^* mice fed a high-fat diet. Glutathione is oxidized to glutathione disulfide (GSSG) to compensate for oxidative stress. Our analysis shows that GSSG levels were decreased in *Thbs1^−/−^* mice fed a high-fat diet. This could be mediated by the activity of glutathione reductase and glutathione peroxidase. Potentially due to an overall decrease in glutathione production or an increase in the reduced form. Another critical component of regulating redox homeostasis is the cysteine/cystine ratio, a lower percentage marker of oxidative stress in the clinic [35]. Cystine, the oxidized form of cysteine, accumulated in *Thbs1^−/−^* mice fed a high-fat diet. Increased cysteine levels have been shown to cause pro-inflammatory signaling in cardiovascular disease, and lower cysteine levels occur in patients with NAFLD [35,36]. Methionine and S-methyl cysteine were elevated in *Thbs1^−/−^* mice. *Thbs1^−/−^* mice also had reduced levels of 4-hydroxy-nonenal-glutathione (HNE-GSH) when fed a high-fat diet compared to WT animals (−4.32 log2-fold in WT vs. −6.64 log2-fold in *Thbs1^−/−^*). Methionine is an essential amino acid, and methionine deficiency induces steatosis in animals [37,38]. Methionine is used to synthesize glutathione and metabolizes it to S-adenosylmethionine, an important methyl donor. Increased homocysteine levels are associated with increased oxidative stress, inflammation, and NAFLD [39]. These changes in metabolites suggest TSP1-dependent regulation of redox stress responses.

The progression of NAFLD is often associated with global and hepatic insulin resistance, which alters hepatic gluconeogenesis and glucose flux [40]. Glycolytic metabolites, including lactate, were downregulated in WT mice fed a high-fat diet compared to those fed a low-fat diet, suggesting a shift in glucose metabolism due to fat consumption (Figure 2C). *Thbs1^−/−^* mice had increased pentose phosphate metabolites, including ribulose/xylulose 5-phosphate, 6-phosphogluconate, and threitol, suggesting a potential shift in glucose metabolism toward the pentose phosphate pathway, which could perturb regulation of NADPH levels.

**Figure 4:**
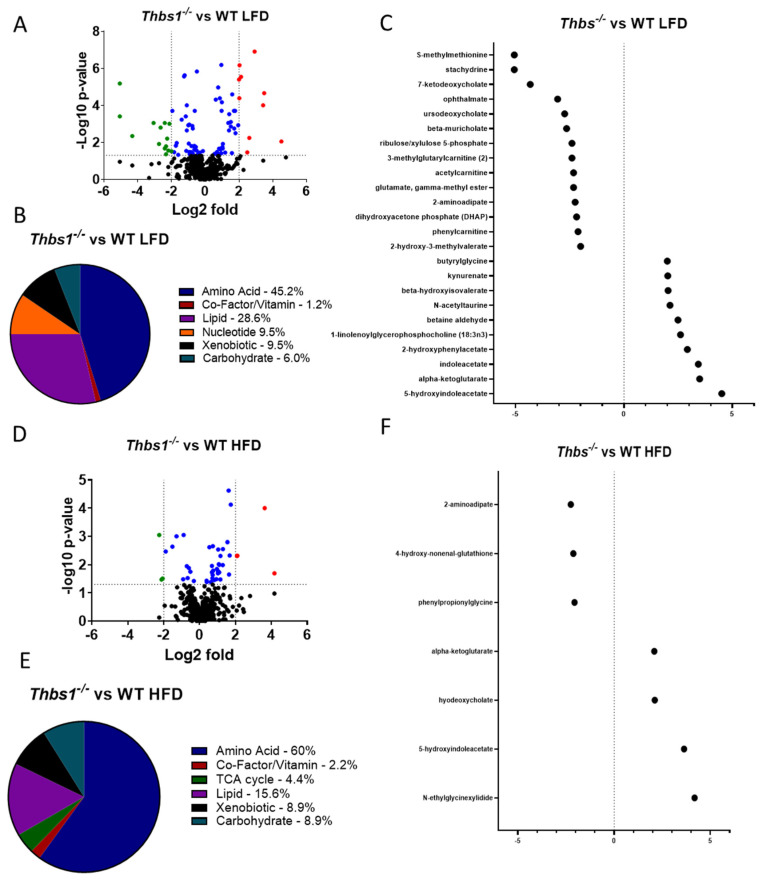
Lack of thrombospondin-1 (TSP1) alters liver metabolism. (**A**) Volcano plot showing the number of metabolites regulated by TSP1 deficiency in animals fed a low-fat diet (LFD). Downregulated < −2-fold in green; upregulated > 2-fold in red; significantly altered between −2 and 2 in blue. (**B**) Pie chart depicting metabolites that exhibited significant TSP1-dependent changes in mice fed an LFD. (**C**) Dot plot showing significantly regulated metabolites < −2- and > 2-fold in TSP1 deficient mice fed an LFD. (**D**) Volcano plot shows the number of metabolites regulated by TSP1 deficiency in animals fed a high-fat diet (HFD). Downregulated < −2-fold in green. (**E**) Pie chart depicting metabolites that exhibited significant TSP1-dependent changes in mice fed an HFD. (**F**) Dot plot showing significantly regulated metabolites < −2- and > 2-fold in TSP1-deficient mice fed an HFD. N = 8/each group.

**Differential regulation of dietary metabolites by TSP1.** To determine whether diet consumption is differentially regulated in WT and *Thbs1^−/−^* mice, we compared liver metabolites from each genotype in high-fat and low-fat diet-fed mice. We observed 89 metabolites that differed in livers of *Thbs1^−/−^* vs. WT mice fed a low-fat diet (Figure 4A and Supplementary Table S3). About 45% of these are amino acid metabolites, with 25 metabolites elevated in *Thbs1^−/−^* compared to WT (Figure 4A,B). Pathway enrichment analysis indicated that glutamate, alanine, and aspartate metabolic pathways are the most regulated when comparing *Thbs1^−/−^* to WT. However, the most significantly upregulated metabolite was 5-hydroxy indole acetate, a tryptophan metabolite generated from serotonin (Figure 4C and Supplementary Table S3). Cholangiocytes, stellate liver cells, and microbes in the gut can produce serotonin from tryptophan [30,41]. Increases in serotonin metabolites, including 5-hydroxy indole acetate, are associated with NAFLD development [42]. The tryptophan metabolites indole acetate and kynurenine were also among the most elevated in *Thbs1^−/−^* livers compared to WT livers, indicating a general TSP1-dependent regulation of tryptophan metabolism (Figure 4C).

Lipid metabolites account for about 26% of differentially regulated metabolites between *Thbs1^−/−^* and WT mice fed a low-fat diet. The most significantly elevated metabolite was 1-linolenoyl glycerophosphocholine, a biomarker for developing insulin resistance, glucose intolerance, and type II diabetes [43,44]. *Thbs1^−/−^* mice had decreased levels of the primary bile acids cholate and beta-muricholate as well as two secondary bile acids relative to WT mice, supporting a role for TSP1 in regulating cholesterol homeostasis [45].

Several TCA metabolites were elevated in *Thbs1^−/−^* mice, most prominently α-ketoglutarate, with an over 3 log2-fold increase compared to WT mice fed the same low-fat diet. α-Ketoglutarate is a central intermediate metabolite of the TCA cycle, plays a vital role in regulating energy metabolism, and has been linked to obesity-associated liver pathology (Supplementary Table S2) [46,47,48].

Approximately 9% of the metabolites regulated by genotype on a low-fat diet were nucleotide metabolites. All nucleotide metabolites were significantly depleted in *Thbs1^−/−^* mice relative to WT. This may reflect decreased oxidative stress associated with a low-fat diet but may implicate TSP1 deficiency in the capacity to overcome liver injury.

**Figure 5:**
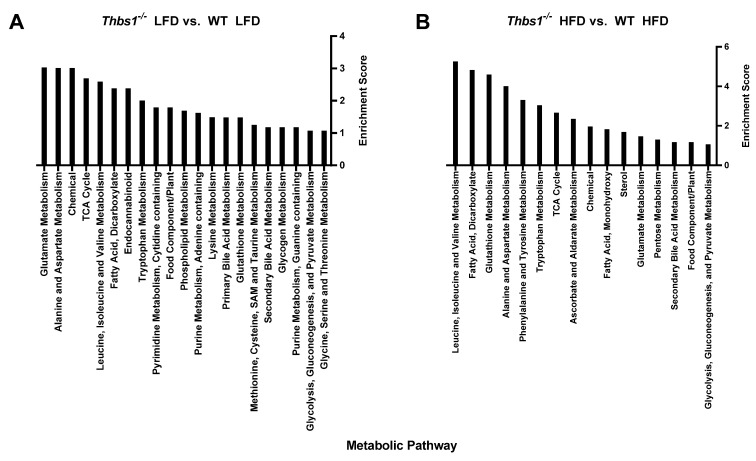
Metabolic pathway enrichment analysis. (**A**) Comparing TSP1-dependent metabolic pathways in mice fed a low-fat diet (LFD). (**B**) Comparing TSP1-dependent metabolic pathways in mice fed a high-fat diet (HFD).

Comparing *Thbs1^−/−^* to WT mice fed a high-fat diet, we observed 45 differentially regulated metabolites, with the majority (60%) occurring in amino acid metabolites (Figure 4D,E and Supplementary Table S4). Similar to what was observed in the low-fat diet, 5-hydroxy indole acetate and indole acetate were also elevated in the *Thbs1^−/−^* animals fed a high-fat diet (Figure 4F). Pathway enrichment analysis indicated leucine, isoleucine, and valine metabolism regulation when comparing *Thbs1^−/−^* to WT mice fed a high-fat diet, thus suggesting differences compared to low-fat diet-fed mice (Figure 5A). Consistent with the animals fed a low-fat diet, α-ketoglutarate remained significantly elevated in *Thbs1^−/−^* mice fed a high-fat diet, supporting the role of TSP1 in regulating this TCA cycle metabolite (Figure 4F). As observed when feeding *Thbs1^−/−^* a high-fat diet, we observed an over log2-fold reduction in 4-hydroxy-nonenal-glutathione in *Thbs1^−/−^* mice fed a high-fat diet compared to WT. Pathway enrichment analysis also shows that glutathione metabolism is significantly regulated in *Thbs1^−/−^* mice (Figure 5B). These alterations suggest that *Thbs1^−/−^* mice may handle oxidative stress associated with high-fat diet consumption differently from WT.

Results from this global biochemical profiling study revealed metabolic differences in liver tissue when comparing WT and *Thbs1^−/−^* mice fed a low-fat or high-fat diet, with distinct clustering of metabolites based on genotype-specific handling of the diets. Exposure to a high-fat diet was associated with differential changes in lipid metabolism, TCA cycle intermediates, lipid-derived eicosanoids, amino acid, and protein metabolism compared to mice of the identical genotypes fed the low-fat diet. Notably, several of these pathways have been previously implicated in developing liver injury, including NAFLD. NAFLD is a spectrum of diseases in individuals who do not over-consume alcohol [49]. NASH is an advanced presentation of the disease. It occurs when inflammation and hepatocellular ballooning with or without fibrosis in addition to the lipid accumulation and can advance to liver cirrhosis [50]. Despite the relatively high prevalence of fatty liver disease with obesity, only a subset of the population progresses to inflammation and chronic liver disease [51]. Histopathology is essential in evaluating NAFLD/NASH, which occurs clinically when at least 5% of hepatocytes display lipid accumulation [49]. However, there is a need for designing non-invasive approaches and uncovering potential biomarkers to detect and stage NAFLD/NASH progression.

Obesity, the pathological excess of body fat resulting from excess caloric intake, is a significant risk factor in developing NAFLD [52]. Therefore, in order to determine whether TSP1 is implicated in regulating metabolism relevant to NAFLD, we performed a liver metabolomics analysis in WT and *Thbs1^−/−^* animals fed low-fat or high-fat diets. Adipose tissue regulates energy balance and glucose homeostasis by secreting adipokines [53]. Increased adiposity occurs during obesity and can lead to anatomic and functional abnormalities that manifest as altered adipokine production and other changes [54,55,56]. A study on high-fat diet-induced obesity in a TSP1-deficient mouse model showed that TSP1 deficiency improved glucose–insulin homeostasis while decreasing adipose macrophage accumulation and inflammation [57]. TSP1 mRNA expression and serum TSP1 were increased in rats fed high-fat diets. These animals also had lower insulin-stimulated glucose uptake and higher TSP1 expression in cultured adipocytes [58].

Furthermore, in a murine model of diet-induced NASH, *Thbs1^−/−^* mice fed a choline-deficient L-amino acid-defined high-fat diet were protected against developing some of the characteristics of NASH including fibrosis, suggesting that TSP1 is critical for the development of NASH [12]. In the same study, *Thbs1^−/−^* mice exhibited decreased serum lipid levels and decreased mRNA levels of tumor necrosis factor-alpha (TNFα) and TGFβ1, which are associated with NAFLD progression [12]. Serum TSP1 and TSP1 mRNA levels are increased in patients with NAFLD and other liver diseases and were positively associated with increasing severity of liver steatosis [23,59]. Administration of recombinant TSP1 or the CD36-binding TSP1 mimetic ABT-526 inhibited liver steatosis in murine models of diet-induced obesity [23]. These studies show the involvement of TSP1 in NAFLD and the potential role of the TSP1-CD36 signaling axis.

When we compared the impact of consumption of a high-fat diet within each genotype, we observed a similar proportion of regulated metabolites associated with fatty acid metabolism. However, consuming a high-fat diet suppresses the number of genotype-dependent liver metabolites, whereas the number of high-fat diet-dependent metabolites is greater in the *Thbs1^−/−^* mice. This is evidenced by the significant number of metabolites with an over 2-fold regulation in *Thbs1^−/−^* mice and may further support studies suggesting no significant differences in lipid accumulation in the livers of *Thbs1^−/−^* mice. Still, pathway enrichment analysis suggested high regulation of sterols in *Thbs1^−/−^* mice, which may have implications for converting cholesterol to bile acids and liver homeostasis. Compared to WT mice fed a high-fat diet, *Thbs1^−/−^* mice had increased tauro-beta-muricholate. This bile acid can act as an agonist of the Farnesoid X receptor (FXR) superfamily, regulating bile acid homeostasis and lipid metabolism, reducing bile acids, and contributing to hepatic steatosis [27,28]. When we compared the effect of diet between each genotype, we observed that the absence of TSP1 leads to the regulation of a larger proportion of amino acid metabolites. Previous studies examining transcriptional differences in murine models of NAFLD between WT and *Thbs1^−/−^* mice suggested pathways related to amino acid metabolism as necessary in regulating NAFLD-mediated liver damage [12]. Our analysis indicated differences in the regulation of tryptophan metabolism, which has been implicated in liver tissue integrity. Specifically, the microbiota-derived tryptophan metabolite indole acetate [31,60] was found to be upregulated in WT mice fed a high-fat diet compared to *Thbs1^−/−^*. Administration of this metabolite in obese mice protects against NAFLD by reducing lipid accumulation, inflammation, and oxidative stress [31]. Therefore, the relative reduction of indole acetate in *Thbs1^−/−^* supports that the loss of TSP1 may be detrimental to liver homeostasis during obesity-induced stress. Another metabolite associated with obesity severity that was upregulated in *Thbs1^−/−^* regardless of diet was α-ketoglutarate; this TCA metabolite is linked to the severity of obesity and steatohepatitis [46,47]. We had previously reported the relative metabolite intensity of α-ketoglutarate in WT and *Thbs1^−/−^* and had observed that it also remains elevated in the *Thbs1^−/−^ Apc^Min/+^*, suggesting that TSP1 may be a key modulator of this metabolite. Loss of the TSP1 receptor CD47 alters several metabolic pathways that consume or produce α-ketoglutarate [61], suggesting that the observed alteration in *Thbs1^−/−^* and *Thbs1^−/−^ Apc^Min/+^* mice may be mediated in part by the loss of CD47 signaling. This could be relevant to the pathogenesis of NASH because *Cd47^−/−^* mice chronically fed a high-fat diet develop more severe hepatic steatosis and fibrosis [62]. In contrast, *Cd47^−/−^* mice fed a high-fat diet for 16 weeks were protected against hepatic lipid accumulation and had decreased inflammation [63], suggesting a possible temporal implication of TSP1 signaling.

Another pathway commonly regulated in *Thbs1^−/−^* mice was glutathione metabolism, which is well known to be a regulator of oxidative stress [64]. A critical metabolite in this pathway, 4-hydroxy-nonenal-glutathione is reduced in the *Thbs1^−/−^* mice; 4-hydroxy-nonenal-glutathione is a hepatic metabolite formed by conjugating 4-hydroxynonenal (HNE) with glutathione and is a known marker of oxidative stress [65]. It is a breakdown product of lipid peroxidation that reacts with DNA. Its conjugation with glutathione prevents the formation of DNA adducts, thus preventing DNA damage [66]. This represents another mechanism by which the loss of TSP1 could mediate liver damage during oxidative stress and NAFLD progression [67]. This is also consistent with other reports demonstrating that inhibition of TSP1 is associated with decreased/reduced glutathione activity in a model of acetaminophen-induced liver toxicity [67]. This glutathione regulation was associated with lowering nuclear factor-erythroid 2-related factor 2 (Nrf2), which is critical in mediating antioxidant signaling [67]. Regulation of glutathione by TSP1 under conditions of redox stress could also be a CD47-dependent process, because CD47-deficient T cells were protected from the depletion of reduced glutathione induced by exposure to ionizing radiation and maintained higher levels of metabolites required for glutathione biosynthesis [61].

The mechanisms of liver pathology associated with NAFLD are complex. The current understanding of pathogenesis invokes a “multiple parallel hits” mechanism involving the gut, adipose tissue, immune system, and liver contributing to a cycle of inflammation and fibrosis and eventually damage [68]. It is clear that many factors beyond fat accumulation in the liver drive the progression of NAFLD. Our data and others support the notion that TSP1 may be critical in regulating these processes. The paradoxical effects may be due to the spatiotemporal expression of this matricellular protein; while it may be increased in circulation in patients with NAFLD, the absence of TSP1 may also elicit harmful metabolic mechanisms that compromise liver tissue integrity. The difference may also be due to the source of TSP1; monocyte/macrophage populations release TSP1, contributing to insulin resistance and the progression of fatty liver and fibrosis, and macrophages of TSP1-deficient mice had reduced inflammatory phenotype macrophages and macrophage activation [57,60,68]. The role of TSP1 in macrophage activation is partially mediated through TSP1 interacting with CD36, suppressing *Smpdl3b* expression, amplifying toll-like receptor 4, nuclear factor-κB, and TNF-α expression [60,69]. Therefore, the metabolic changes observed in liver tissue could be mediated by the effects of TSP1 release from macrophages on hepatocyte metabolism. Furthermore, the differential effects may be due to differences in microbiota; we observed the regulation of several secondary metabolites that depend on microbial processing. The role of TSP1 in modulating the microbiome remains to be defined and needs further study.

## 3. Methods

**Sample processing.** The samples analyzed in this study were processed using the metabolon^®^ platform as reported previously [10]. Scaled intensities were reported previously as a measurement of metabolite levels [10]. Following log transformation and imputation of missing values, if any, with the minimum observed value for each compound, ANOVA contrasts were used to identify biochemicals that differed significantly between experimental groups. An estimate of the false discovery rate (q-value) is calculated both unadjusted and adjusted *p*-values, and scale intensities for each metabolite can be found in DOI: 10.1038/oncsis.2016.37(accessed 1 September 2022).

**Data set.** The data set used in this study is based on the data published previously by Soto-Pantoja et al. in [10] with the purpose of reanalyzing the data to determine genotype and diet effects between WT and *Thbs1^−/−^* mice [10]. Briefly, mice were fed an AIN-76A (5% fat) or a Western diet (#D12079B) with 21% fat beginning at weaning (Research Diets https://researchdiets.com/) (accessed 1 September 2022) [10]. Livers of 12-week-old mice were harvested and subjected to tissue metabolomics using metabolon’s platform as described previously [10].

**Discrete correlate summation and principal component analysis**. One matrix of log correlation ratios (log_cr_) was calculated, as described previously [70], for all metabolites in both conditions. Briefly, the correlation matrices were transformed into linear probability matrices using Student’s *t*-distribution for (n-2) degrees of freedom, returning two tails. A matrix of log_cr_ was calculated (absolute value of the logarithm of the ratio of each comparison’s *p*-value) from the matrices of metabolites from WT and *Thbs1^−/−^* mice fed a low-fat diet and mice fed a high-fat diet. The correlation matrix of this log_cr_ matrix was calculated, and each column was totaled. The original log_cr_ matrix was sorted in descending order based on these totals, establishing a clear clustering pattern. The upper cluster of this original matrix was determined by the totals of each variable column. The original matrix total array average was calculated right to left (RL-OriMaTArA). The last column in the contiguous block (LaCoCoB_25_) reading right to left at which the RL-OriMaTArA is less than or equal to the 25th percentile [Q1, black sums and RL-OriMaTArA, (Appendix A Figure A1 and Figure A2)] for the array was determined. If this point is in the rightmost 33% of the array (purple fraction, SF1 and 2), clusters were defined below using Q1. If this point is in the center 33% of the array (green fraction, SF1 and 2), clusters were determined below the median (Q2, white sums and RL-OriMaTArA, SF1 and 2). The value used as cut-off is Q*. Going left to right, the variables before the first value in the matrix total array less than Q* are in the DCS cluster. This distinction allows the discrete evaluation of clustering quality for the matrix, as is evident in SF1 and 2. For a matrix with a LaCoCoB_25_ in the green fraction with little or diffuse clustering, the higher Q2 cut-off captures the smaller clustered segment. A matrix indicated by a purple fraction LaCoCoB_25_, the lower Q1 cut-off allows for the more distinct and robust clustering.

**Volcano Plots**: The volcano plots were calculated plotting the negative logarithm base 10 of the *p*-value on the y-axis and the logarithm of the fold-change of two conditions in the x-axis. The cut-off for significance is *p* < 0.05. The negative logarithm base 10 of adjusted q-values was also calculated and is included in the Supplementary Tables S1–S4.

**Pathway enrichment analysis:** We performed a pathway enrichment analysis (https://portal.metabolon.com) using the formula (k/m)/((n − k)(N − m). Where K equals the number of significant metabolites; m equals the total number of detected metabolites in a pathway; n equals the total number of significant metabolites; and N equals the total number of metabolites.

## Data Availability

Data sets analyzed can be found at https://www.nature.com/articles/oncsis201637 (accessed on 1 September 2022).

## Supplementary Materials

The following supporting information can be downloaded at: https://www.mdpi.com/article/10.3390/metabo12111036/s1, Supplementary Tables S1–S4. Data set can be found at DOI:10.1038/oncsis.2016.37.

## Abbreviations

The following abbreviations are used in this manuscript:

MDPI	Multidisciplinary Digital Publishing Institute
DCS	Discrete Correlation Summation
DOAJ	Directory of open access journals
HFD	High-fat diet
LFD	Low-fat diet
NAFLD	Nonalcoholic fatty liver disease
NASH	Nonalcoholic steatohepatitis
PCA	Principal Component Analysis
Thsbs1/TSP1	Thrombospondin-1
Versus	vs.
WT	Wild type
