# Supplementary material for: Discrete Correlation Summation Clustering Reveals Differential Regulation of Liver Metabolism by Thrombospondin-1 in Low-Fat and High-Fat Diet-Fed Mice
Source: Metabolites. 2022 Oct 28;12(11):1036. doi: 10.3390/metabo12111036 (PMC9697255; doi:10.3390/metabo12111036)
Supplement: Supplementary file 1 [file metabolites-12-01036-s001.zip › metabolites-1892940-supplementary.pdf]

Table S1. Significantly regulated liver metabolites between Wild type high-fat diet vs wild type low-fat diet

Metabolite	Log2fold	-LOG(p value,10)	-LOG(q value,10)
indoleacetate	2.887525271	4	2.161151
eicosapentaenoate (EPA; 20:5n3)	2.17951105	3.698970004	2
hexenedioylcarnitine*	2.021479727	3.22184875	1.806875
N-oleoyltaurine	1.752748591	2.744727495	1.555955
2-hydroxyphenylacetate	1.63691458	3.698970004	2.045757
myristoleate (14:1n5)	1.510961919	4.318586888	2.267606
10-nonadecenoate (19:1n9)	1.378511623	3.522878745	1.966576
15-methylpalmitate (isobar with 2-methylpalmitate)	1.372952098	4.086790343	2.161151
stearidonate (18:4n3)	1.310340121	2.585026652	1.514279
myristate (14:0)	1.275007047	4	2.161151
isobutyrylglycine	1.232660757	1.307153081	0.737075
N1-Methyl-2-pyridone-5-carboxamide	1.22650853	1.818156412	1.014574
N-palmitoyltaurine	1.195347598	1.806875402	1.014574
3-hydroxy-2-ethylpropionate	1.189033824	2.823908741	1.59176
10-heptadecenoate (17:1n7)	1.176322773	3.045757491	1.742321
phenylcarnitine*	1.169925001	2.853871964	1.59176
xanthurenate	1.169925001	1.314258261	0.737075
pentadecanoate (15:0)	1.124328135	4.498899639	2.267606
alpha-tocopherol	1.111031312	1.673664139	0.931814
3-methylcrotonylglycine	1.042644337	1.671620397	0.931814
docosahexaenoate (DHA; 22:6n3)	1.014355293	2.27572413	1.29243
beta-hydroxyisovalerate	0.992768431	2.638272164	1.519993
methylsuccinate	0.97819563	3	1.701147
docosapentaenoate (n3 DPA; 22:5n3)	0.97819563	1.66756154	0.931814
margarate (17:0)	0.925999419	2.214670165	1.244125
laurate (12:0)	0.887525271	2.585026652	1.514279
2-methylbutyrylglycine	0.86393845	1.906578315	1.051098
cis-urocanate	0.807354922	1.382999659	0.762708
eicosenoate (20:1n9 or 11)	0.757023247	1.537602002	0.854182
palmitoleate (16:1n7)	0.659924558	1.809668302	1.014574
N-palmitoyl-sphingosine*	0.650764559	1.54668166	0.854182
N-palmitoyl glycine	0.641546029	1.325138859	0.737075
myo-inositol	0.584962501	1.804100348	1.014574
nicotinamide adenine dinucleotide (NAD+)	0.575312331	1.30980392	0.737075
2-hydroxyisobutyrate	0.516015147	2.301029996	1.299296
adenosine 5'-monophosphate (AMP)	0.50589093	2.022276395	1.117475

histidine	0.454175893	2.721246399	1.555955
flavin adenine dinucleotide (FAD)	0.432959407	1.625251654	0.895854
cysteine-glutathione disulfide	0.321928095	1.677780705	0.931814
valine	0.214124805	1.356547324	0.751536
threonine	0.189033824	1.54515514	0.854182
beta-hydroxyisovaleroylcarnitine	-0.340075442	1.443697499	0.806597
carnitine	-0.377069649	1.450996738	0.807154
palmitoyl-linoleoyl-glycerophosphocholine (2)*	-0.454031631	1.73754891	0.96457
oxalate (ethanedioate)	-0.59946207	1.316052869	0.737075
arabonate	-0.64385619	1.41453927	0.782779
1-stearoylglycerol (1-monostearin)	-0.736965594	1.504455662	0.832092
lactate	-0.76121314	2.619788758	1.519993
hydroxybutyrylcarnitine*	-0.785875195	1.449771647	0.807154
3-hydroxyoctanoate	-0.810966176	1.70333481	0.937794
docosapentaenoate (n6 DPA; 22:5n6)	-0.836501268	2.040958608	1.117475
malonylcarnitine	-0.836501268	1.591760035	0.868061
5-HETE	-0.888968688	1.328827157	0.737075
xylulose	-0.943416472	2.677780705	1.528708
valerate	-0.943416472	1.369572125	0.753748
cytidine diphosphate	-1	1.549750892	0.854182
campesterol	-1.217591435	3.15490196	1.806875
caproate (6:0)	-1.217591435	1.452225295	0.807154
erythritol	-1.251538767	2.408935393	1.379864
folate	-1.286304185	1.53165267	0.853562
methionine sulfoxide	-1.358453971	1.886056648	1.041914
prostaglandin F2alpha	-1.434402824	2.619788758	1.519993
dehydroascorbate	-1.473931188	2.769551079	1.559091
15-HETE	-1.514573173	2.161150909	1.201349
1-stearoylglycerophosphocholine (18:0)	-1.556393349	1.946921557	1.071604
2-oleoylglycerol (2-monoolein)	-1.59946207	2.142667504	1.191789
S-methylmethionine	-1.689659879	3.397940009	1.966576
alpha-hydroxyisovaleroyl carnitine*	-1.689659879	2.408935393	1.379864
1-oleoylglycerophosphocholine (18:1)	-1.689659879	1.356547324	0.751536
12,13-DiHOME	-1.836501268	1.542118103	0.854182
prostaglandin E2	-1.943416472	2.327902142	1.315155
6-oxopiperidine-2-carboxylic acid	-1.943416472	1.341035157	0.740884
1-arachidonoylglycerophosphocholine (20:4n6)*	-2	1.924453039	1.057496
1-stearoylglycerophosphoglycerol	-2	1.490797478	0.824488
pyridoxal	-2.120294234	3.15490196	1.806875
13-HODE + 9-HODE	-2.251538767	2.823908741	1.59176

maltotriose	-2.251538767	1.974694135	1.077275
1-linoleoylglycerophosphocholine (18:2n6)	-2.251538767	1.812479279	1.014574
stachydrine	-2.251538767	1.480172006	0.820736
2-palmitoylglycerophosphocholine*	-2.321928095	1.749579998	0.9694
ophthalmate	-2.836501268	2.568636236	1.511449
hyodeoxycholate	-3.184424571	3.522878745	1.982967
adenylosuccinate	-3.184424571	1.387216143	0.762708
7-ketodeoxycholate	-3.64385619	1.844663963	1.015023
4-hydroxy-nonenal-glutathione	-4.321928095	3.301029996	1.882729
maltose	-4.321928095	1.879426069	1.041914
1-palmitoylglycerophosphoglycerol*	-4.64385619	5.608994282	3.09691
1-methylnicotinamide	-4.64385619	1.568636236	0.854182
xanthine	-5.64385619	2.031517051	1.117475
tauro-beta-muricholate	-6.64385619	2.065501549	1.127844

Table S2. Significantly regulated liver metabolites between Thbs1−/− Low-fat diet vs Thbs1−/− high-fat diet

**Metabolite**	**Log2fold**	**-LOG(p value,10)**	**-LOG(q value,10)**
N-ethylglycinexylidide	4.171527106	1.694648631	1.189095719
hexenedioylcarnitine*	3.973611276	10.25217662	7.994090554
dihydroxyacetone phosphate (DHAP)	3.711494907	2.721246399	1.931814138
7-alpha-hydroxy-3-oxo-4-cholestenoate (7-Hoca)	3.667892125	4.592949185	3.22184875
phenylcarnitine*	3.480265122	9.891739495	7.809780392
Isobar: fructose 1,6-diphosphate, glucose 1,6-diphosphate, myo-inositol 1,4 or 1,3-diphosphate	3.401903472	2.060480747	1.431798276
3-methylglutarylcarnitine (2)	3.396433531	5.471237514	4
lidocaine	3.198494154	2.22184875	1.542118103
stachydrine	3.085764554	1.795880017	1.250263684
eicosapentaenoate (EPA; 20:5n3)	2.889473543	6.498844847	4.784838492
ribulose/xylulose 5-phosphate	2.731183242	2.37675071	1.653647026
pentadecanoate (15:0)	2.464668267	13.02227639	10.46113734
15-methylpalmitate (isobar with 2-methylpalmitate)	2.432959407	6.699187206	4.91825675
17-methylstearate	2.292781749	1.899629455	1.299296283
glutamate, gamma-methyl ester	2.163498732	2.167491087	1.515700161
myristoleate (14:1n5)	2.15704371	8.042775942	6.085741488

cystine	2.056583528	1.391473966	0.993962045
10-nonadecenoate (19:1n9)	1.981852653	4.697474267	3.301029996
ursodeoxycholate	1.914564523	2.086186148	1.446116973
10-heptadecenoate (17:1n7)	1.883620816	4.402666877	3.096910013
hyocholate	1.839959587	1.338187314	0.963371105
beta-muricholate	1.655351829	1.886056648	1.294992041
3-methylglutarylcarnitine (1)	1.63691458	2.886056648	2.017728767
laurate (12:0)	1.599317794	7.007278629	5.147154182
stearidonate (18:4n3)	1.580145484	4.744462174	3.301029996
myristate (14:0)	1.575312331	5.529339648	4
6-phosphogluconate	1.560714954	2.568636236	1.793174124
margarate (17:0)	1.459431619	3.301029996	2.301029996
docosapentaenoate (n3 DPA; 22:5n3)	1.448900951	2.769551079	1.954677021
cytidine-3'-monophosphate (3'-CMP)	1.378511623	3.397940009	2.356547324
gamma-glutamylglutamine	1.333423734	1.385102784	0.990124366
N6-succinyladenosine	1.304511042	2.920818754	2.026872146
squalene	1.280956314	2.443697499	1.692503962
docosahexaenoate (DHA; 22:6n3)	1.214124805	3.522878745	2.397940009
phenol sulfate	1.182692298	1.515700161	1.087777943
3-hydroxy-2-ethylpropionate	1.15704371	3.096910013	2.173925197
methylsuccinate	1.10433666	3.698970004	2.602059991
oleate (18:1n9)	1.049630768	2.795880017	1.954677021
palmitoyl-oleoyl-glycerophosphocholine (1)*	1.007195501	2.318758763	1.609064893
oleoyl-linoleoyl-glycerophosphocholine (2)*	0.97819563	1.790484985	1.247183569
10-undecenoate (11:1n1)	0.956056652	1.679853714	1.181114585
1-docosahexaenoylglycerol	0.918386234	1.777283529	1.243363892
glutamate	0.895302621	2.744727495	1.946921557
cis-vaccenate (18:1n7)	0.887525271	1.76700389	1.241088108
19,20-DiHDPA	0.879705766	2.795880017	1.954677021
2-docosahexaenoylglcyerol*	0.879705766	1.605548319	1.14691047
aspartate	0.871843649	3.096910013	2.173925197
2'-deoxycytidine 5'-monophosphate	0.855989697	2.026872146	1.40560745
stearoyl-arachidonoyl-glycerophosphoserine (1)*	0.839959587	1.3400838	0.963371105
tyrosine	0.831877241	2.769551079	1.954677021
tetradecanedioate	0.748461233	1.4710833	1.053056729
inositol 2-phosphate (I2P)	0.739848103	1.581698709	1.126098402
glycerol	0.695993813	1.679853714	1.181114585
threitol	0.687060688	1.804100348	1.250263684

S-methylcysteine	0.678071905	2.050609993	1.42365865
caprate (10:0)	0.678071905	1.779891912	1.243363892
palmitoleate (16:1n7)	0.669026766	1.612610174	1.14691047
5-dodecenoate (12:1n7)	0.641546029	1.522878745	1.087777943
glutamine	0.632268215	3.698970004	2.619788758
indoleacetate	0.613531653	1.514278574	1.087777943
ornithine	0.575312331	1.882728704	1.294992041
beta-hydroxyisovalerate	0.575312331	1.49757288	1.075204004
tagatose	0.555816155	1.395773947	0.994819487
5-hydroxyindoleacetate	0.526068812	1.543633967	1.099086932
N-acetylmethionine	0.516015147	2.167491087	1.515700161
tryptophan	0.495695163	3.522878745	2.468521083
proline	0.495695163	3.045757491	2.142667504
methionine	0.485426827	2.455931956	1.705533774
asparagine	0.475084883	5.26256085	3.698970004
3-hydroxylaurate	0.454175893	1.469800302	1.053056729
thymine	0.443606651	3.397940009	2.356547324
cholesterol	0.443606651	3.045757491	2.142667504
alpha-tocopherol	0.422233001	1.605548319	1.14691047
deoxycarnitine	0.422233001	1.41453927	1.00656377
5-methyl-2'-deoxycytidine	0.411426246	1.744727495	1.223298816
N1-methylguanosine	0.378511623	2.119186408	1.4710833
histidine	0.367371066	1.844663963	1.266802735
serine	0.263034406	3.522878745	2.397940009
isoleucine	0.250961574	1.795880017	1.250263684
phenylalanine	0.250961574	1.517126416	1.087777943
threonine	0.23878686	2.193820026	1.518557371
lysine	0.22650853	1.93930216	1.329754147
3-indoxyl sulfate	0.214124805	1.413412695	1.00656377
biopterin	-0.304006187	1.442492798	1.029653124
fumarate	-0.358453971	1.632644079	1.159893906
glutathione, oxidized (GSSG)	-0.377069649	1.40560745	1.001740662
palmitoyl-linoleoyl-glycerophosphocholine (2)*	-0.454031631	1.91721463	1.313363731
cytidine	-0.454031631	1.640164518	1.163675884
arabonate	-0.535331733	1.341035157	0.963371105
cytidine 5'-monophospho-N-acetylneuraminic acid	-0.556393349	1.698970004	1.189095719
N-acetylarginine	-0.556393349	1.555955204	1.107348966
thiamin (Vitamin B1)	-0.621488377	2.301029996	1.605548319
xylulose	-0.64385619	1.327902142	0.956244873
beta-hydroxyisovaleroylcarnitine	-0.666576266	3.301029996	2.301029996

salicylate	-0.666576266	1.879426069	1.294992041
2-hydroxystearate	-0.689659879	1.812479279	1.250263684
pipecolate	-0.76121314	2.180456064	1.515700161
2-hydroxypalmitate	-0.785875195	2.070581074	1.437707136
valerylglycine	-0.836501268	1.735182177	1.216811309
propionylglycine	-0.836501268	1.348721986	0.965772739
1-stearoylglycerol (1-monostearin)	-0.862496476	2.346787486	1.621602099
adrenate (22:4n6)	-0.862496476	1.575118363	1.122628654
adenosine 3',5'-diphosphate	-0.888968688	1.517126416	1.087777943
8-hydroxyoctanoate	-0.971430848	1.321481621	0.952335805
beta-sitosterol	-1	2.721246399	1.931814138
1-stearoylglycerophosphoserine*	-1.089267338	1.669586227	1.178486472
folate	-1.217591435	1.657577319	1.172630727
kynurenine	-1.251538767	2.769551079	1.954677021
5-HETE	-1.251538767	1.372634143	0.983802646
threonate	-1.286304185	1.378823718	0.986741335
2-linoleoylglycerol (2-monolinolein)	-1.321928095	1.348721986	0.965772739
butyrylglycine	-1.358453971	3.397940009	2.356547324
docosapentaenoate (n6 DPA; 22:5n6)	-1.358453971	2.292429824	1.600326279
indole-3-carboxylic acid	-1.358453971	1.872895202	1.292429824
kynurenate	-1.434402824	2.387216143	1.655607726
1-palmitoylglycerophosphoinositol*	-1.434402824	1.806875402	1.250263684
methionine sulfoxide	-1.514573173	1.642065153	1.163675884
hexanoylglycine	-1.556393349	2.387216143	1.655607726
nicotinamide riboside	-1.556393349	1.806875402	1.250263684
1-stearoylglycerophosphoglycerol	-1.689659879	1.655607726	1.172630727
1-palmitoylglycerophosphoglycerol*	-1.736965594	3.397940009	2.356547324
15-HETE	-1.736965594	2.30980392	1.609064893
prostaglandin F2alpha	-1.785875195	4	2.769551079
N-acetylphenylalanine	-1.785875195	2.236572006	1.555955204
6-oxopiperidine-2-carboxylic acid	-1.785875195	1.93930216	1.329754147
3-hydroxyoctanoate	-1.836501268	5.51778407	4
alpha-hydroxycaproate	-1.836501268	2.886056648	2.017728767
sphingosine	-1.836501268	1.835647144	1.262807357
glutarate (pentanedioate)	-1.888968688	4	2.657577319
N-formylphenylalanine	-1.888968688	1.673664139	1.179142011
2-hydroxyadipate	-1.943416472	1.512861625	1.087777943
N-acetyltaurine	-2.058893689	1.701146924	1.189095719
caproate (6:0)	-2.184424571	4	2.744727495
pyridoxal	-2.395928676	3.698970004	2.568636236
campesterol	-2.556393349	6.361291155	4.705291342

N-acetylisoleucine	-2.556393349	2.30980392	1.609064893
2-oleoylglycerol (2-monoolein)	-2.556393349	1.76700389	1.241088108
3-hydroxypyridine sulfate	-2.736965594	4.483253149	3.15490196
prostaglandin E2	-2.836501268	4.382632657	3.096910013
2-aminoheptanoate	-2.836501268	3.045757491	2.142667504
1-linolenoylglycerophosphocholine (18:3n3)*	-3.058893689	2.886056648	2.017728767
13-HODE + 9-HODE	-3.321928095	4.244452894	2.958607315
1-arachidonoylglycerophosphocholine (20:4n6)*	-3.321928095	2.920818754	2.026872146
2-palmitoleoylglycerophosphocholine*	-3.473931188	2.167491087	1.515700161
phenylpropionylglycine	-3.836501268	3.22184875	2.27572413
1-linoleoylglycerophosphocholine (18:2n6)	-3.836501268	3.096910013	2.173925197
1-oleoylglycerophosphocholine (18:1)	-4.058893689	2.585026652	1.818156412
cinnamoylglycine	-4.058893689	2.508638306	1.744727495
1-methylnicotinamide	-4.058893689	1.617982957	1.148741651
1-stearoylglycerophosphocholine (18:0)	-4.64385619	3.301029996	2.318758763
2-stearoylglycerophosphocholine*	-4.64385619	3.301029996	2.318758763
1-palmitoleoylglycerophosphocholine (16:1)*	-5.058893689	3.045757491	2.142667504
2-palmitoylglycerophosphocholine*	-5.64385619	4.228463961	2.958607315
4-hydroxy-nonenal-glutathione	-6.64385619	4.077264165	2.853871964
7-hydroxycholesterol (alpha or beta)	-6.64385619	2.602059991	1.826813732

Table S3. Significantly regulated liver metabolites between Wild type vs *Thbs1*$^{-/-}$ low-fat diet

Metabolite	Log2fold	-LOG(p value,10)	-LOG(q value,10)
5-hydroxyindoleacetate	4.510329019	2.045757491	1.130181792
alpha-ketoglutarate	3.49057013	4.655745307	3.096910013
indoleacetate	3.428946345	4	2.522878745
2-hydroxyphenylacetate	2.922197848	6.900802945	4.314849663
1-linolenoylglycerophosphocholine (18:3n3)*	2.608809243	2.236572006	1.303643611
betaine aldehyde	2.49057013	1.457174573	0.746661995
N-acetyltaurine	2.121015401	5.536450404	3.698970004
beta-hydroxyisovalerate	2.03562391	6.164848669	4.056011125
kynurenate	2.021479727	4.380322435	2.958607315

butyrylglycine	2	5.39184852	3.698970004
hexanoylglycine	1.952333566	2.920818754	1.882728704
isovalerylcarnitine	1.790772038	2.494850022	1.54668166
3-hydroxy-2-ethylpropionate	1.769771739	3.698970004	2.494850022
3-hydroxypyridine sulfate	1.718087584	2.744727495	1.759450752
isovalerylglycine	1.700439718	3.698970004	2.48148606
2-hydroxyglutarate	1.599317794	4.587253937	3.096910013
N-octanoylglycine	1.580145484	1.415668776	0.721475035
4-methyl-2-oxopentanoate	1.516015147	3.096910013	1.943095149
3-methylcrotonylglycine	1.516015147	2.853871964	1.832682665
2-methylbutyrylglycine	1.422233001	3.522878745	2.301029996
3-methyl-2-oxovalerate	1.40599236	2.638272164	1.669586227
valerylglycine	1.367371066	3.045757491	1.928117993
guanidinoacetate	1.182692298	1.444905551	0.745210313
isovalerate	1.042644337	1.913640169	1.030584088
N1-Methyl-2-pyridone-5-carboxamide	1.03562391	1.663540266	0.870632404
N-palmitoyltaurine	1.021479727	1.744727495	0.919373513
taurine	0.98550043	4.163176037	2.769551079
gamma-aminobutyrate (GABA)	0.970853654	3.698970004	2.443697499
fumarate	0.956056652	6.181061867	4.056011125
anthranilate	0.948600847	1.404503778	0.720789487
3-hydroxyoctanoate	0.90303827	1.607303047	0.831797253
N-oleoyltaurine	0.871843649	1.663540266	0.870632404
2-hydroxyisobutyrate	0.86393845	4.382224077	2.958607315
methylmalonate (MMA)	0.847996907	1.366531544	0.710188161
5-oxoproline	0.815575429	1.653647026	0.867420152
alanine	0.765534746	4.965331444	3.397940009
putrescine	0.695993813	1.472370099	0.74860515
succinate	0.641546029	1.397940009	0.720789487
malate	0.622930351	4.308945044	2.886056648
methylsuccinate	0.555816155	1.374687549	0.712422191
serine	-0.13606155	1.527243551	0.779630368
2'-deoxycytidine	-0.321928095	1.449771647	0.745210313
maltotetraose	-0.415037499	1.37675071	0.712422191
asparagine	-0.49410907	5.825591268	4
3-hydroxylaurate	-0.514573173	1.555955204	0.794254459
deoxycarnitine	-0.556393349	1.749579998	0.919373513
glutamate	-0.59946207	1.701146924	0.886056648
glutamine	-0.621488377	3.698970004	2.468521083
3-aminoisobutyrate	-0.621488377	1.742321425	0.919373513
adenosine	-0.64385619	1.809668302	0.9476909

caprate (10:0)	-0.64385619	1.605548319	0.831797253
ornithine	-0.736965594	2.744727495	1.759450752
guanosine 3'-monophosphate (3'-GMP)	-0.736965594	1.397940009	0.720789487
lactate	-0.76121314	2.886056648	1.869666232
2'-deoxycytidine 5'-monophosphate	-0.810966176	1.795880017	0.941194513
erythritol	-0.836501268	1.465973894	0.74860515
glycerophosphoethanolamine	-0.836501268	1.373659633	0.712422191
adenosine 2'-monophosphate (2'-AMP)	-0.862496476	1.32330639	0.684449466
4-guanidinobutanoate	-0.915935735	1.361510743	0.710188161
threitol	-0.943416472	2.958607315	1.906578315
3-methylglutarylcarnitine (1)	-0.943416472	1.477555766	0.74860515
2-hydroxybutyrate (AHB)	-0.971430848	1.931814138	1.037157319
N6-succinyladenosine	-1	2.920818754	1.879426069
pentadecanoate (15:0)	-1.089267338	4	2.568636236
cytidine-3'-monophosphate (3'-CMP)	-1.089267338	2.638272164	1.669586227
glycerophosphorylcholine (GPC)	-1.120294234	1.53313238	0.779630368
19,20-DiHDPA	-1.152003093	3.397940009	2.193820026
tyrosine	-1.217591435	5.620132711	3.698970004
aspartate	-1.251538767	5.558477674	3.698970004
5-ketogluconate	-1.395928676	3.22184875	2.096910013
2-hydroxyadipate	-1.59946207	1.321481621	0.684449466
6-oxopiperidine-2-carboxylic acid	-1.689659879	1.962573502	1.057495894
lidocaine	-1.736965594	1.815308569	0.9476909
cholate	-1.836501268	1.467245621	0.74860515
hexenedioylcarnitine*	-1.943416472	3.698970004	2.494850022
2-hydroxy-3-methylvalerate	-2	1.515700161	0.774432287
phenylcarnitine*	-2.120294234	3	1.910094889
dihydroxyacetone phosphate (DHAP)	-2.184424571	1.554395797	0.794254459
2-aminoadipate	-2.251538767	2.200659451	1.27572413
glutamate, gamma-methyl ester	-2.321928095	1.782516056	0.936291441
acetylcarnitine	-2.321928095	1.356547324	0.710188161
3-methylglutarylcarnitine (2)	-2.395928676	3.045757491	1.928117993
ribulose/xylulose 5-phosphate	-2.395928676	1.671620397	0.870632404
beta-muricholate	-2.64385619	2.795880017	1.787812396
ursodeoxycholate	-2.736965594	1.910094889	1.030584088
ophthalmate	-3.058893689	3.045757491	1.928117993
7-ketodeoxycholate	-4.321928095	2.337242168	1.391473966
stachydrine	-5.058893689	5.177518086	3.522878745
S-methylmethionine	-5.058893689	3.397940009	2.229147988

Table S4. Wild type vs *Thbs1*$^{-/-}$ high-fat diet

Metabolite	Log2fold	-LOG(p value,10)	-LOG(q value,10)
N-ethylglycinexylidide	4.171527106	1.694648631	0.486516043
5-hydroxyindoleacetate	3.626439137	4	1.774690718
hyodeoxycholate	2.10433666	2.318758763	0.841939206
alpha-ketoglutarate	2.080657663	2.30980392	0.841939206
3-hydroxy-2-ethylpropionate	1.735522177	4.12346464	1.774690718
guanidinoacetate	1.673556424	2.327902142	0.841939206
isovalerylcarnitine	1.641546029	1.651695137	0.456677099
beta-hydroxyisovalerate	1.618238656	4.621474918	1.920818754
6-phosphogluconate	1.550900665	2.795880017	0.93330145
3-methyl-2-oxovalerate	1.298658316	2.552841969	0.91150953
isovalerylglycine	1.292781749	1.987162775	0.591251394
2-hydroxyphenylacetate	1.163498732	2.30980392	0.841939206
indoleacetate	1.15704371	1.473660723	0.372634143
5-oxoproline	1.097610797	1.991399828	0.591251394
4-hydroxyphenylpyruvate	1.09085343	1.725842151	0.489320969
taurocyamine	1.070389328	2.017728767	0.591251394
alpha-hydroxyisovalerate	1.03562391	2.537602002	0.91150953
4-methyl-2-oxopentanoate	1.028569152	1.769551079	0.489320969
dehydroascorbate	1.021479727	1.694648631	0.486516043
isovalerate	0.963474124	1.844663963	0.524764777
putrescine	0.933572638	1.484126156	0.372634143
7-alpha-hydroxy-3-oxo-4-cholestenoate (7-Hoca)	0.815575429	1.453457337	0.366228654
xylulose	0.757023247	1.787812396	0.489320969
2-hydroxyisobutyrate	0.739848103	2.657577319	0.911863911
3-methyl-2-oxobutyrate	0.731183242	1.634512015	0.452840879
gamma-aminobutyrate (GABA)	0.713695815	1.515700161	0.372634143
methylsuccinate	0.687060688	1.954677021	0.591251394
2-hydroxyglutarate	0.687060688	1.728158393	0.489320969
pyruvate	0.622930351	1.381951903	0.335546071
alanine	0.545968369	2.619788758	0.911863911
fumarate	0.40053793	1.386158178	0.335546071
p-cresol sulfate	0.389566812	1.432973634	0.356054087
glutathione, oxidized (GSSG)	-0.321928095	1.42136079	0.355462942
cytidine 5'-monophospho-N-acetylneuraminic acid	-0.514573173	1.749579998	0.489320969
aspartate	-0.59946207	1.882728704	0.54515514
kynurenine	-0.666576266	1.525783736	0.372634143
2-hydroxystearate	-0.713118852	1.946921557	0.591251394

2-hydroxypalmitate	-0.888968688	3.045757491	1.061480275
1-stearoylglycerophosphoethanolamine	-0.915935735	1.48148606	0.372634143
5-ketogluconate	-1.286304185	3	1.061480275
6-oxopiperidine-2-carboxylic acid	-1.514573173	2.638272164	0.911863911
2-hydroxyadipate	-1.888968688	2.468521083	0.883060353
phenylpropionylglycine	-2.058893689	1.507239611	0.372634143
4-hydroxy-nonenal-glutathione	-2.120294234	1.469800302	0.372634143
2-aminoadipate	-2.251538767	3.045757491	1.061480275
